# Long Term Natural History Data in Ambulant Boys with Duchenne Muscular Dystrophy: 36-Month Changes

**DOI:** 10.1371/journal.pone.0108205

**Published:** 2014-10-01

**Authors:** Marika Pane, Elena Stacy Mazzone, Serena Sivo, Maria Pia Sormani, Sonia Messina, Adele D′Amico, Adelina Carlesi, Gianluca Vita, Lavinia Fanelli, Angela Berardinelli, Yvan Torrente, Valentina Lanzillotta, Emanuela Viggiano, Paola D′Ambrosio, Filippo Cavallaro, Silvia Frosini, Andrea Barp, Serena Bonfiglio, Roberta Scalise, Roberto De Sanctis, Enrica Rolle, Alessandra Graziano, Francesca Magri, Concetta Palermo, Francesca Rossi, Maria Alice Donati, Michele Sacchini, Maria Teresa Arnoldi, Giovanni Baranello, Tiziana Mongini, Antonella Pini, Roberta Battini, Elena Pegoraro, Stefano Previtali, Claudio Bruno, Luisa Politano, Giacomo P. Comi, Enrico Bertini, Eugenio Mercuri

**Affiliations:** 1 Department of Paediatric Neurology, Catholic University, Rome, Italy; 2 Biostatistics Unit, Department of Health Sciences, University of Genoa, Genoa, Italy; 3 Department of Neurosciences, Psychiatry and Anaesthesiology, University of Messina, Messina, Italy; 4 Department of Neurosciences, Unit of Neuromuscular and Neurodegenerative Disorders, Bambino Gesù Children's Hospital, Rome, Italy; 5 Child Neurology and Psychiatry Unit, “Casimiro Mondino” Foundation, Pavia, Italy; 6 Dino Ferrari Centre, Neuroscience Section, Department of Pathophysiology and Transplantation, University of Milan, Neurology Unit, Ca' Granda Ospedale Maggiore Policlinico, Milan, Italy; 7 Neuromuscular Disease Unit, Giannina Gaslini Institute, Genoa, Italy; 8 Dipartimento di Medicina Sperimentale, Seconda Università di Napoli, Napoli, Italy; 9 Department of Developmental Neuroscience, Stella Maris Institute, Pisa, Italy; 10 Department of Neurosciences, University of Padua, Padua, Italy; 11 Child Neurology and Psychiatry Unit, Maggiore Hospital, Bologna, Italy; 12 Neuromuscular Center, San Giovanni Battista Hospital, University of Turin, Turin, Italy; 13 Metabolic and Neuromuscular Unit, Meyer Hospital, Florence, Italy; 14 Developmental Neurology Unit, Neurological Institute Carlo Besta, Milan, Italy; 15 Department of Neurology, San Raffaele Scientific Institute, Milan, Italy; The Hospital for Sick Children, Canada

## Abstract

The 6 minute walk test has been recently chosen as the primary outcome measure in international multicenter clinical trials in Duchenne muscular dystrophy ambulant patients. The aim of the study was to assess the spectrum of changes at 3 years in the individual measures, their correlation with steroid treatment, age and 6 minute walk test values at baseline. Ninety-six patients from 11 centers were assessed at baseline and 12, 24 and 36 months after baseline using the 6 minute walk test and the North Star Ambulatory Assessment. Three boys (3%) lost the ability to perform the 6 minute walk test within 12 months, another 13 between 12 and 24 months (14%) and 11 between 24 and 36 months (12%). The 6 minute walk test showed an average overall decline of −15.8 (SD 77.3) m at 12 months, of −58.9 (SD 125.7) m at 24 months and −104.22 (SD 146.2) m at 36 months. The changes were significantly different in the two baseline age groups and according to the baseline 6 minute walk test values (below and above 350 m) (p<0.001). The changes were also significantly different according to steroid treatment (p = 0.01). Similar findings were found for the North Star Ambulatory Assessment. These are the first 36 month longitudinal data using the 6 minute walk test and North Star Ambulatory Assessment in Duchenne muscular dystrophy. Our findings will help not only to have a better idea of the progression of the disorder but also provide reference data that can be used to compare with the results of the long term extension studies that are becoming available.

## Introduction

In the last few years the rapidly increasing number of potentially effective therapeutical approaches for patients affected by Duchenne muscular dystrophy (DMD) has highlighted the need for improving clinical trial design. A lot of attention has been devoted to natural history studies and to the identification of possible trajectories of progression of the disease [1]–[5]. The 6 minute walk test (6MWT) is currently being used as the primary outcome measure in most of the ongoing studies as it provides a global assessment of functional mobility, endurance, and ability to walk [6]–[9]. The North Star Ambulatory Assessment (NSAA) a motor functional scale specifically developed for DMD, is also often used as a secondary measure providing additional information on several functional aspects that reflect everyday life activities. Both measures fit the construct for DMD, have proved to be reliable, have been validated and used in multicentric longitudinal studies [8]–[12] and reflect clinically meaningful activities.

We have recently reported the changes observed on both 6MWT and NSAA over 12 and 24 months in ambulant DMD boys, identifying slopes of deterioration at different ages (above and below 7 years) and cut off points predicting loss of ambulation [9], [13]. Other studies have also highlighted other criteria for identifying trajectories of progression, such as baseline values above or below 350 meters (m) [1]–[3]. These findings have been largely used for defining inclusion and stratification criteria in the recently planned clinical trials. As part of an international effort, several workshops with advocacy groups and regulatory agents have recently further explored how the combination of all the available natural history data may improve other aspects of clinical trial design, in terms of expected results and power of the study.

In the present study we report the results at 36 months using the same assessments. The aim of the present study was to assess the spectrum of changes in the individual measures, and their correlation not only with age and treatment with steroids, but also to other variables, such as baseline values, in accordance with recent literature.

## Subjects and Methods

The study is a longitudinal multicentric cohort study involving 11 tertiary neuromuscular centers in Italy. Patients were recruited between January 2008 and June 2010 and followed for at least three years with the last follow up visit performed in August 2013. The study was approved by the Ethical Committees of all the participating centers (Catholic University, Rome; University of Messina; Ospedale Bambino Gesù, Rome; Istituto Mondino, Pavia; Gaslini Institute, Genoa; Besta Institute, Milan; Stella Maris Institute, Pisa; Ospedale Maggiore, Bologna; University of Napoli; University of Turin; University of Padua; University of Milano). As the assessments were already part of the clinical routine in all centers, with the approval of the Ethics Committees, verbal consent to record the anonymized data in a database was obtained by the parents for the boys under age.

Patient inclusion criteria at baseline were: genetically proven DMD diagnosis, patient still ambulant and able to walk independently for at least 75 m, no severe or moderate learning difficulties or behavioral problems. Genetic and treatment information were collected and classified following the criteria used in our previous study [9]. All patients attending the 11 participating centers who fulfilled the inclusion criteria were enrolled in the study. As part of the routine assessments in all centers patients are seen at least once every 12 months and all centers performed at each visit the NSAA followed by the 6MWT. Data were collected from the first assessment after recruitment (baseline) from the 12, 24 and 36-month follow up assessment.

As previously reported [19] as the various centers used different types of steroids (deflazacort and prednisone) and had different regimes, we broadly subdivided our cohort into: a) no steroids: this included boys who had never been on steroids and others who had used them for less than a year and had stopped treatment at least one year before the study; b) intermittent regime, patients who had been, at the time of the study, on alternate days or alternate weeks or 10 days on/10 days off of either.75 mg of prednisone or.9 mg/kg/day of deflazacort for at least a year; c) daily regime, patients who had been, at the time of the study, on daily treatment of.75 mg of prednisone or.9 mg/kg/day of deflazacort for over a year, also including those in whom the dose had not been always completely adjusted to the current weight. A small number of patients who took deflazacort on alternate days but with a dose of approximately 2 mg/kg were also included in this group as their monthly dose was similar if not higher to those with a standard daily dose of steroids.

Details of the training for the physiotherapists involved in the study and of the interobserver reliability for NSAA among the centers have already been reported [8]–[10].

### Standard protocol approvals, registrations, and patient consents

As this was an observational study, requiring non invasive procedures of assessment that were used routinely in all centers and did not require extra visits to the centres, no informed consent was required as the data were analyzed anonymously. Parents of participants (all our patients were minor/children) and patients were informed that the data collected as part of our routine clinical assessment were going to be used anonymously for an observational study defining natural history of the diseases and they all gave verbal consent. Written consent was not required as the data were analyzed anonymously. The Ethical committee from Catholic University, Rome approved this procedure. All clinical investigation was conducted according to the principles expressed in the Declaration of Helsinki.

### 6MWT

6MWT was performed in all DMD ambulant boys older than 5 according to the ATS guidelines [14], modified by having two examiners, one recording time and distances and one staying close to the patient for safety issues.

### NSAA

The scale consists of 17 items, ranging from standing (item 1) to running (item 17) and includes several items assessing abilities that are necessary to remain functionally ambulant, items assessing abilities, such as head raise and standing on heels that can be partly present in the early stages of the disease and a number of activities such as hopping, or running that are generally never fully achieved in untreated DMD boys but that have been found in those treated with daily steroids.

Each item can be scored on a 3 point scale using simple criteria: 2 -Normal achieves goal without any assistance; 1 -Modified method but achieves goal independent of physical assistance from another person; 0 - Unable to achieve independently.

A total score can be achieved by summing the scores for all the individual items. The score can range from 0, if all the activities are failed, to 34, if all the activities are achieved.

### Statistical analysis

The dependence of NSAA, 6MTW, 10 m, and Gowers values on time was evaluates using a repeated measure analysis of variance, adjusting for baseline age (<7 years vs ≥7 years), baseline 6MWT (<350 m vs ≥350 m) and the use of steroids at baseline (no vs alternate or continuous).

Correlations were evaluated by the Spermann rank correlation coefficients.

## Results

Of the previously reported 113 patients [13] who fulfilled the inclusion criteria and entered the study, 96 also had an assessment at 36 months. One died, two were lost at follow up and the other 14 entered interventional clinical trials.

All the tests were performed safely without any mayor fall during the performance of the individual measures. Of the 96 boys, 42 were on daily (mean age 8.6 years, mean baseline 6MWT 373.04 m) and 49 on intermittent steroids (mean age 8.1 years, mean baseline 6MWT 384.28 m). The remaining 5 were not on steroids.

### 6MWT

Three boys (3%) lost the ability to perform the test within 12 months, another 13 between 12 and 24 months from baseline (14%) and other 11 between 24 and 36 months (12%) (table 1). The 6MWD showed an average overall decline of −15.8 (SD 77.3) m at 12 months, of −58.9 (SD 125.7) m at 24 months and −104.22 (SD 146.2) m at 36 months, that was statistically significant (p for time trend<0.001).

**Table 1:** 36-month changes in the cohort subdivided according to age (above or below 7 years) and 6MWT (above or below 350 m).

Age		6MWT		NSAA	
		<350 m (n = 34)	≥350 m (n = 62)	<350 m (n = 34)	≥350 m (n = 62)
		(n = 9)	(n = 19)	(n = 9)	(n = 19)
	Age range	5–6.9	5–6.9		
<7 years (n = 28)	(mean(SD)) (in years)	(5.8(0.72))	(6.16(0.45))		
	Mean (SD) (in m)	−49.3 (173.0)	19.1 (76.8)	−7.77 (10.99)	−3.5 (6.2)
	N losing ambulation	n = 2	n = 1		
		(n = 25)	(n = 43)	(n = 27)	(n = 43)
	Age range	7–12.4	7–12.8		
≥7 years (n = 68)	(mean(SD)) (in years)	(9.87(2.06))	(8.9(1.43))		
	Mean (SD) (in m)	−199.2 (121.8)	−115.0 (136.5)	−12.74 (4.94)	−7.7 (8.0)
	N losing ambulation	n = 16	n = 5		

**Figure 1:**
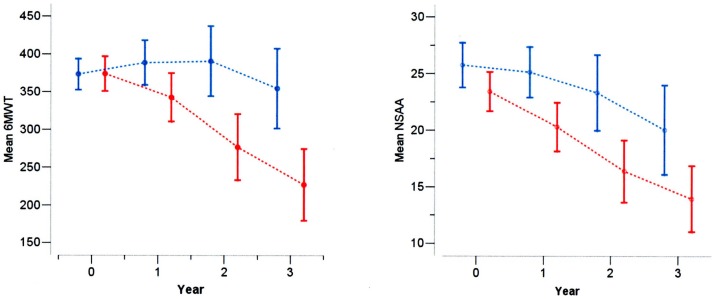
6MWT (left pannel) and NSAA (right pannel) at baseline, 1, 2 and 3 years in DMD boys, below (red) and above (blu) the age of 7 years.

The changes were significantly different in the two baseline age groups (test for interaction, p<0.001): boys below 7 years remained on the average stable with also a slight increase at 12 months (mean 6MWT change  =  +18.5 (SD = 60.7) m) and also at 24 months (mean 6MWT change  = +34.11 (SD = 88.8) m), with a small decrease at 36 months (mean 6MWT change  = −2.9 (SD = 117.7) m). Boys above 7 years had a decrease of about 30 m at 12 months (mean 6MWT change  = −29.9 (SD = 79.3) m), about 100 m at 24 (mean 6MWT change  = −97.3 (SD = 118.9) m) and about 150 m at 36 months (mean 6MWT change  = −146.0 (SD = 146.2) m). (Fig. 1a)

**Figure 2:**
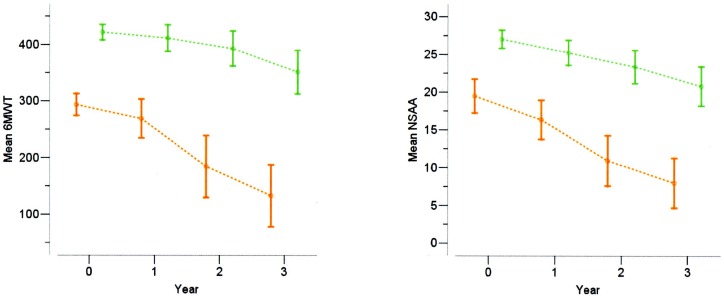
6MWT (left pannel) and NSAA (right pannel) at baseline, 1, 2 and 3 years in DMD boys, below (green) and above (orange) 350 m of 6MWD.

The 6MWT decreases also more rapidly according to the baseline value (above or below 350 m) (p for interaction<0.001): boys with a baseline 6MWT<350 m had a mean 36 month decrease of 160 (SD = 150) m, while boys with a baseline 6MWT≥350 m had a 36 month decrease of 74 (SD = 135) m. (Fig. 2a)

**Figure 3:**
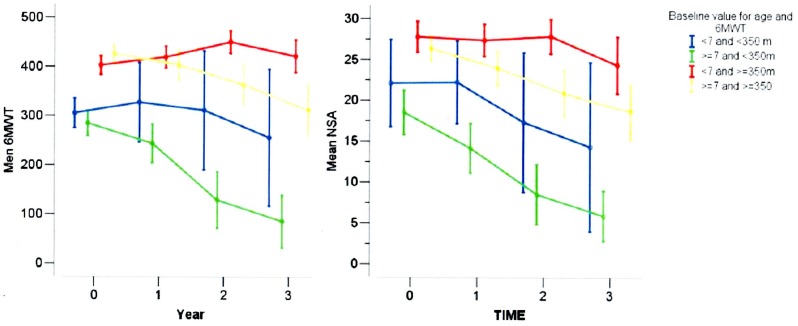
6MWT (left pannel) and NSAA (right pannel) at baseline, 1, 2 and 3 years in DMD boys, subdivided according to a combination of age (below and above 7 years) and distance (below and above 350 m of 6MWD).

The changes were significantly different in the two baseline age groups (test for interaction, p<0.001) and according to the 6MWT baseline value (above or below 350 m) (p for interaction<0.001). Therefore patients were classified in four groups, as those of age <7 years and 6MWT baseline <350 m, age ≥7 years and 6MWT baseline <350, age <7 years and 6MWT baseline ≥350 m, and age ≥7 years and 6MWT baseline ≥350 m. The 6MWT changes were significantly different among the 4 groups (p for time x group interaction<0.001). Values of 6MWT changes at each time point for each subgroup are reported in Fig. 3a and in the table S1.

The changes were also significantly different according to steroid treatment (p for interaction = 0.01): boys who had no treatment had a decrease over 36 month of −216.8 (SD = 84.9) m, boys on alternate treatment of −128.3 (SD = 161.5) m and boys on continuous treatment of −75.0 (SD = 129.6) m.

### NSAA

There was an increasing average decline of −2.29 (SD 3.84) at 12 months, −5.50 (SD 6.79) at 24 months and −8.23 at 36 months (SD = 7.86). The changes were significantly different in the two baseline age groups (test for interaction, p<0.001): boys below 7 years had a small change over the first year (mean NSAA change  = −0.25 (SD = 3.2)), and further decrease at 24 months (mean NSAA change  = −1.5 (SD = 6.3)) and at 36 months (mean NSAA change  = −4.9 (SD = 8.1)). Boys above 7 years had a decrease of about 3 points at 12 months (mean NSAA change  = −3.1, SD = 3.8), higher than 7 years points at 24 months (mean NSAA change  = −7.15, SD = 6.31) and higher than 9 points at 36 months (mean NSAA change  = −9.5, SD = 7.4). (Fig. 1b)

The NSAA decreases also more rapidly according to the 6MWT baseline value (<350 m or ≥350 m) (p for interaction = 0.006): boys with a baseline 6MWT <350 m had a mean 36-month NSAA decrease of 11.5 (SD = 7.1), while boys with a baseline 6MWT ≥350 m had a 36-month decrease of 6.5 (SD = 7.7) m. (Fig. 2b)

The NSAA changes were significantly different in the two baseline age groups (test for interaction, p<0.001) and according to the 6MWT baseline value (<350 m or ≥350 m) (p for interaction<0.001). Values of NSAA changes at each time point for each group are reported in Fig. 3b and in the table S1.

The changes were also significantly different according to steroid treatment (p for interaction = 0.03): boys who had no treatment had a decrease over 36 months of −15.3 (SD = 5.4), boys on alternate treatment of −10.0 (SD = 8.0) and boys on continuous treatment of −6.2 (SD = 7.3).

### Correlations of changes over 36 months

Table 2 shows the correlation between the changes observed on the 6MWT and the NSAA and between the individual measures and age.

**Table 2:** Correlation between the changes at 3 years observed on the 6MWT (D6MWT3) and the NSAA (DNS3) and between the individual measures and age.

			DNS3	D6MTW3	Age
Spearman's rho	DNS3	Correlation Coefficient	1.000	.775**	−.340**
		Sig. (2-tailed)	.	.000	.001
		N	95	95	95
	D6MTW3	Correlation Coefficient	.775**	1.000	−.533**
		Sig. (2-tailed)	.000	.	.000
		N	95	96	96
	Age	Correlation Coefficient	−.340**	−.533**	1.000
		Sig. (2-tailed)	.001	.000	.
		N	95	96	96

**. Correlation is significant at the 0.01 level (2-tailed).

## Discussion

This is the first study reporting 36-month longitudinal natural history data using the 6MWT and NSAA, the two outcome measures most used in current clinical studies in DMD. In our previous studies the two measures had a good correlation at baseline, and at 12 and 24 months follow up and this held true even when the 36 month changes were analyzed.

Our findings showed that in the overall cohort 96 boys there was a progressive deterioration that became sharper with each additional year on both measures. We were however also interested in establishing the long term effect of some variables, such as age and values of 6MWD at baseline and steroid treatment, that have been shown to play an important role in the shorter follow up.

In agreement with our 12 and 24 month data [9], [13] showing a difference between the 6MWD in boys below and above the age of 7 years at baseline, we found that this difference was also present at 36 months. As previously reported, those who were younger than 7 years had some improvement in the first 24 months but this was not maintained in the third year.

Those above the age of 7 years showed a progressive deterioration that was much more marked with each increasing year after baseline.

Similar differences between boys below and above 7 years at baseline were observed on the NSAA even though boys below the age of 7 years showed practically stable results in the first 12 months and some mild deterioration in the following two years.

These findings therefore confirm our previous observation [9], [13], [15] that the age of 7 years appears to be crucial also at the 36-month follow up. Recent studies have also suggested that the trajectories of longitudinal changes over 12 months are different in boys who have different baseline 6MWD, especially between those below or above 350 m [1]. This difference was confirmed also in our study, using a larger cohort and a longer follow up. When we subdivided our cohort according to this cut off point, we also found a significant difference between the two groups with a mean 36-month decrease of 160 m (SD = 150) in the boys with a baseline 6MWT< = 350 m and of only 70 m in those above 350 m (p for interaction<0.001). These results were slightly biased by the fact that the whole group of boys with baseline below 350 m also included a number of young boys who at baseline had 6MWD just below 350 m. Approximately half of them improved over the following years because of growth.

The rate of deterioration in the boys above 7 years and below 350 m was therefore higher (−199.24 m) than that reported in the whole cohort with baseline above 350 m (−160 m).

Following recent studies showing the effect of different regimes of steroids on the progression of the disease using the NSAA and the 6MWT, we also observed that the progression of changes over 3 years was also different according to steroid treatment (p for interaction = 0.01) with an obvious difference between those on alternate and those on continuous treatment.

This study provides for the first time longitudinal data using 6MWT and NSAA over a 36-month period, providing the spectrum of changes observed in each measure and the possible effect of a number of variables such as age and 6MWD at baseline and steroids. Our findings suggest that all these criteria should be considered at the time of designing new clinical trials data as they influence the trajectories of progression. A better knowledge of these aspects will help to power the study more appropriately and to define inclusion and stratification criteria and the duration of the study. This will also depend on the mechanisms of the drug and on the expected results and is particularly relevant for the studies aimed at slowing the progression of the disease. The lack of deterioration in 12 months in young boys with baseline 6MWD above 350 m, suggests for example the need for a longer duration of the study or, following the example of recent ongoing studies, setting up inclusion criteria with only boys above the age of 7 years.

One of the limitations of this study is that until recently height was not consistently measured across the participating centers and we therefore cannot use the Geiger analysis that has previously been used to reduce the effect of age and growth on the 6MWT [16], [17]. Other possible variables, such as the effect of individual groups of mutations [15] and of possible modifying genes, such as osteopontin [18] or LTPB4 [19] are also being currently explored and have not been systematically assessed in our cohort.

Our data will also be useful to compare the 36 month changes observed in our cohort with the long-term results of the first clinical studies on DMD, which are becoming available. Boys involved in the initial 48 week treatment phase, are often enrolled in extension studies that are not placebo controlled. As long term natural history data using the 6MWt have until now not been available, it has not always been easy to draw clear conclusions on the meaning of the long-term changes observed in the extension studies.

The sharper progression with each found in our cohort, especially in the older boys, suggest that the relatively stable results on these measures over two or three years, as reported in some of these studies, may be related to the beneficial efficacy of the drug as this is not common in untreated boys.

## Supporting Information

Table S1 **Changes in 6MWT and North Star Ambulatory Assessment (NSAA) at 12, 24 and 36 months.**
(DOC)
