# Correction: Long Term Natural History Data in Ambulant Boys with Duchenne Muscular Dystrophy: 36-Month Changes

**DOI:** 10.1371/journal.pone.0121882

**Published:** 2015-03-25

**Authors:** The PLOS ONE Staff

There is an error in affiliation 11 for the 31^st^ author, Antonella Pini. The correct affiliation 11 should be: Child Neurology Unit, IRCCS Institute of Neurological Sciences, Bologna, Italy.
