# Correction: Long Term Natural History Data in Ambulant Boys with Duchenne Muscular Dystrophy: 36-Month Changes

**DOI:** 10.1371/journal.pone.0144079

**Published:** 2015-12-04

**Authors:** Marika Pane, Elena Stacy Mazzone, Serena Sivo, Maria Pia Sormani, Sonia Messina, Adele D′Amico, Adelina Carlesi, Gianluca Vita, Lavinia Fanelli, Angela Berardinelli, Yvan Torrente, Valentina Lanzillotta, Emanuela Viggiano, Paola D′Ambrosio, Filippo Cavallaro, Silvia Frosini, Andrea Barp, Serena Bonfiglio, Roberta Scalise, Roberto De Sanctis, Enrica Rolle, Alessandra Graziano, Francesca Magri, Concetta Palermo, Francesca Rossi, Maria Alice Donati, Michele Sacchini, Maria Teresa Arnoldi, Giovanni Baranello, Tiziana Mongini, Antonella Pini, Roberta Battini, Elena Pegoraro, Stefano Previtali, Claudio Bruno, Luisa Politano, Giacomo P. Comi, Enrico Bertini, Eugenio Mercuri

In the legend for Fig 1, blue and red are accidentally inverted. Please view Fig 1 and the correct caption below.

**Fig 1:**
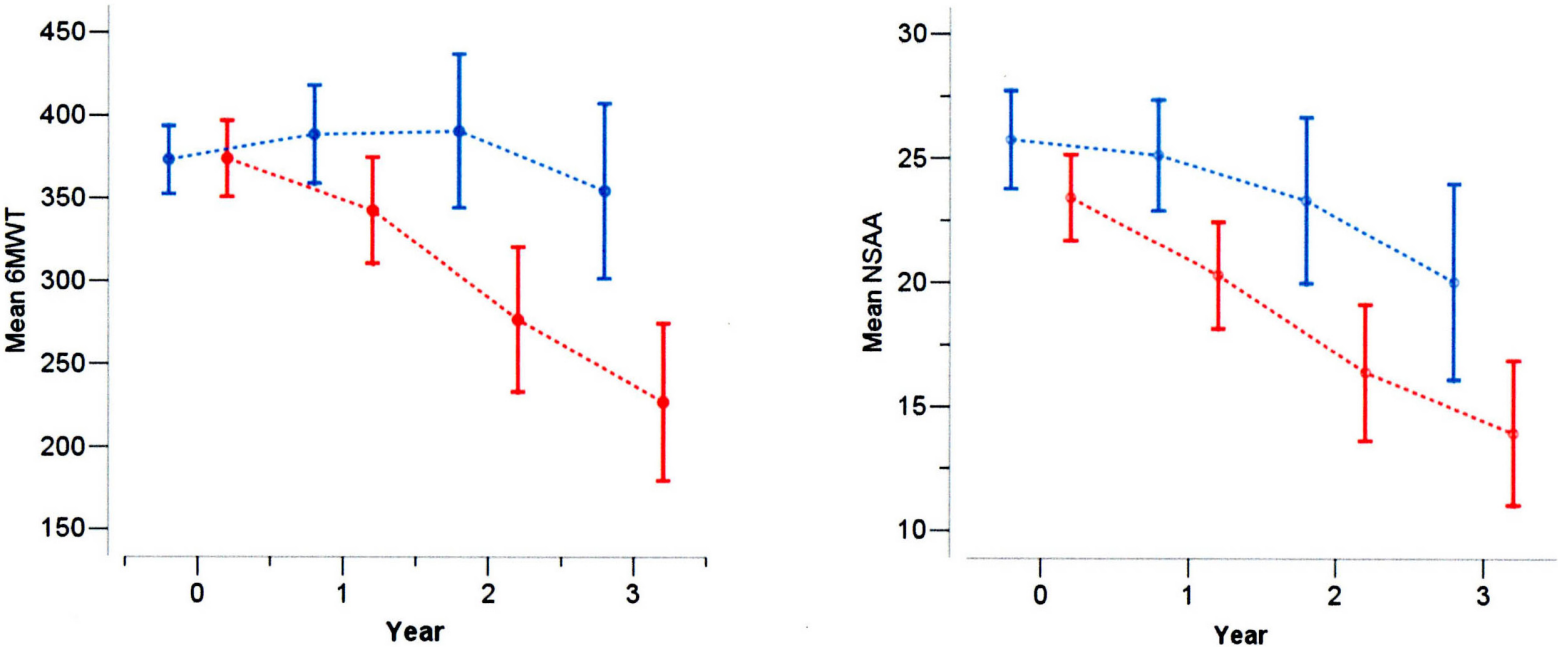
6MWT (left panel) and NSAA (right panel) at baseline, 1, 2 and 3 years in DMD boys, below (blue) and above (red) the age of 7 years.

There is an error in the first sentence of the Results section. The correct sentence is: Of the previously reported 113 patients [13] who fulfilled the inclusion criteria and entered the study, 70 also had an assessment at 36 months and another 26 were new patients, enrolled with the same criteria. Of the 43 patients excluded from the second year, 17 had not reached the 3 year assessment, 4 had assessments at different times but not at 3 years because they entered natural history clinical studies, 5 were younger than 5 years at baseline, 9 were lost at follow up and 8 entered into a clinical study. Two of the 5 patients who were excluded because younger than 5 years had also entered a clinical trial.
