# Supplementary material for: Long Term Natural History Data in Ambulant Boys with Duchenne Muscular Dystrophy: 36-Month Changes
Source: PLoS One. 2014 Oct 1;9(10):e108205. doi: 10.1371/journal.pone.0108205 (PMC4182715; doi:10.1371/journal.pone.0108205)
Supplement: Table S1 — Changes in 6MWT and North Star Ambulatory Assessment (NSAA) at 12, 24 and 36 months. (DOC) [file pone.0108205.s001.doc]

Table S1: Changes in 6MWT and North Star Ambulatory Assessment (NSAA) at 12, 24 and 36 months.

| Group | | △ 6MWT  12 Months | △ 6MWT  24 Months | △ 6MWT  36 Months | △ NSAA  12 months | △ NSAA  24 months | △ NSAA  36 months |
| --- | --- | --- | --- | --- | --- | --- | --- |
| <7y and <350m (n=9) | Mean (SD) | 22 (85.03) | 5.77 (142.81) | 0.11 (3.72) | -4.77(9.4) | -7.77 (10.99) | -49.33 (173) |
| ≥7y and <350m (n=25) | Mean (SD) | -41.32 (88.44) | -156.04 (120.55) | -4.36 (3.42) | -10 (5.3) | -12.64 (4.92) | -199.24 (121.79) |
| <7y and ≥350m (n=19) | Mean (SD) | 16.78 (47.92) | 47.52 (46.46) | -0.42 (3.02) | 0.52 (3.52) | -3.50 (6.15) | 19.10 (76.81) |
| ≥7y and ≥350m (n=43) | Mean (SD) | -23.3 (73.81) | -63.09 (104.91) | -2.41 (3.84) | -5.48 (6.31) | -7.74 (8.04) | -114.97 (136.52) |
| Total (n=96) | Mean (SD) | -15.81 (77.28) | -58.94 (125.76) | -2.29 (3.84) | -5.50 (6.79) | -8.23 (7.86) | -104.22 (146.22) |
